# Fibroblast Growth Factor 2—A Review of Stabilisation Approaches for Clinical Applications

**DOI:** 10.3390/pharmaceutics12060508

**Published:** 2020-06-02

**Authors:** Leah Benington, Gunesh Rajan, Cornelia Locher, Lee Yong Lim

**Affiliations:** 1 Division of Pharmacy, School of Allied Health, University of Western Australia, Crawley 6009, Australia; leah.benington@uwa.edu.au (L.B.); connie.locher@uwa.edu.au (C.L.); 2 Division of Surgery, School of Medicine, University of Western Australia, Crawley 6009, Australia; gunesh.rajan@luks.ch; 3 Department of Otolaryngology, Head & Neck Surgery, Luzerner Kantonsspital, 6000 Luzern, Switzerland

**Keywords:** fibroblast growth factor 2, basic fibroblast growth factor, stabilisation

## Abstract

Basic fibroblast growth factor (FGF)-2 has been shown to regulate many cellular functions including cell proliferation, migration, and differentiation, as well as angiogenesis in a variety of tissues, including skin, blood vessel, muscle, adipose, tendon/ligament, cartilage, bone, tooth, and nerve. These multiple functions make FGF-2 an attractive component for wound healing and tissue engineering constructs; however, the stability of FGF-2 is widely accepted to be a major concern for the development of useful medicinal products. Many approaches have been reported in the literature for preserving the biological activity of FGF-2 in aqueous solutions. Most of these efforts were directed at sustaining FGF-2 activity for cell culture research, with a smaller number of studies seeking to develop sustained release formulations of FGF-2 for tissue engineering applications. The stabilisation approaches may be classified into the broad classes of ionic interaction modification with excipients, chemical modification, and physical adsorption and encapsulation with carrier materials. This review discusses the underlying causes of FGF-2 instability and provides an overview of the approaches reported in the literature for stabilising FGF-2 that may be relevant for clinical applications. Although efforts have been made to stabilise FGF-2 for both in vitro and in vivo applications with varying degrees of success, the lack of comprehensive published stability data for the final FGF-2 products represents a substantial gap in the current knowledge, which has to be addressed before viable products for wider tissue engineering applications can be developed to meet regulatory authorisation.

## 1. Introduction

The fibroblast growth factor (FGF) family of proteins includes signalling proteins secreted by tissues to regulate cell metabolism, proliferation, differentiation, and survival. The biological functions and endogenic roles of FGFs in tissue development and repair have been widely studied. These proteins bind heparin and have broad mitogenic and angiogenic activities, including the regulation of normal cell growth in the epithelium [1], bone [2], soft connective [3,4] and nervous [5] tissues. Not surprisingly, FGFs are regarded as essential components in the construct of functional tissues for tissue engineering applications. The focus of this review is the fibroblast growth factor 2 (FGF-2), also known as basic fibroblast growth factor (bFGF).

FGF-2 was first isolated from the bovine pituitary in 1974 [6] and the first human recombinant FGF-2 was reported in 1988 [7]. The human FGF-2 gene encodes not one protein, but a complex set of isoforms. The secreted isoform has a molecular mass of 18 kg/mol [8] and is a single-chain, non-glycosylated polypeptide with 154 amino acids. The amino acid sequence of human FGF-2 is 99% homologous to that of bovine FGF-2 [9], and it also has high homology with ovine [10] and rodent [11] FGF-2, suggesting strong sequence conservation for structure and function. FGF-2 binds to and activates the FGF receptors mainly via the RAS/MAP kinase pathway to regulate cell proliferation, migration and differentiation, as well as angiogenesis, in a variety of tissues, including skin, blood vessel, muscle, adipose, tendon/ligament, cartilage, bone, tooth and nerve [12,13,14]. These multiple functions make FGF-2 an attractive component for inclusion into wound healing and tissue engineering constructs.

To date, the efficacy of medicinal products incorporating FGF-2 into novel tissue engineering constructs has primarily been investigated using cellular or animal models of human disease [15,16]. The limited clinical translation of these studies may be evidenced by the small number of recently published human clinical trials in the literature [17,18,19,20,21,22,23,24,25,26,27,28,29,30]. Most of these focused on wound healing, with a majority on the repair of traumatic tympanic membrane (TM) perforations by Lou et al. The effectiveness of FGF-2 for the treatment of traumatic tympanic membrane is controversial due to the high rate of spontaneous closure for acute traumatic perforations [31,32]. Indeed, Lou et al. found no significant differences in closure rate and mean closure time for traumatic TM perforations treated with high dose (0.25–0.3 mL of a 21,000 IU/5 mL solution) and low dose (0.1–0.15 mL) FGF-2 [21,33]. Significantly, they have also found no difference between the application of FGF-2 and 0.3% ofloxacin eardrops [34]. This led them to speculate that it was the moist eardrum environment afforded by the eardrop applications that might have aided in shortening the closure time and improve the closure rate of large traumatic TM perforations [35]. In contrast, Kanemaru et al. recorded closure rates of 98.1% in patients with chronic TM perforations treated with FGF-2-soaked gelatin sponges against just 10% in patients treated with saline-soaked sponges [17]. These results, along with a restoration of hearing function and a lack of serious adverse effects, suggest that FGF-2 may offer a significantly less invasive, and a more accessible and safer intervention to conventional surgical repair for chronic TM perforations.

In a clinical trial on chronic skin ulcer, evidence of wound bed healing was observed in 16 of 17 patients treated with a sustained release FGF-2-impregnated collagen/gelatin sponge (CGS) [36]. The CGS was impregnated with a high (14 μg/cm^2^) or low (7 μg/cm^2^) dose of human recombinant FGF-2 and was shown to provide continuous release of the FGF-2 load over a period of at least 10 days. There were no significant differences in efficacy between the high or low dose treatments, and only mild and transient adverse effects were noted in all the patients. The FGF-2 impregnated CGS was also evaluated for use in reconstructive surgical applications [30]. The FGF-2 CGS was applied as the first stage of reconstructive surgery for various acute skin defects including deep dermal burns, facial full-thickness skin defects, and finger amputations, with the second stage of wound closure involving the application of an autologous skin graft, if required. Of the eight patients treated, three did not require the second stage treatment, three required a split-thickness skin graft, and two required a full thickness skin graft to achieve complete healing. Favourable outcomes were observed for all patients in the study, with the FGF-2 CGS treatment providing a minimally invasive alternative to conventional surgical treatments. The clinical data is very promising; however, the practical translation of FGF-2 to clinical use is severely limited by the inherently poor stability of the protein in solution. FGF-2 degrades so rapidly in aqueous media that it is difficult to detect and quantify the bioactive protein in plasma and cell culture samples [37]. In wound healing applications, effective healing requires daily administration of multiple high doses of FGF-2 to compensate for the rapid loss of biological function of the administered FGF-2 at the wound sites [38,39,40].

The aims of this review are to discuss the underlying causes of FGF-2 instability and to provide an overview of the approaches reported in the literature for stabilising FGF-2 that may be relevant for clinical applications. Our view is that FGF-2 must be adequately stabilised in solution to provide a practical, economically feasible and clinically acceptable way forward for its fabrication into wound healing and tissue bioengineering constructs.

## 2. FGF-2 Structure and Stability

The crystal structure of FGF-2 suggests that it is a globular protein with an approximate folded diameter of 4 nm [41]. It has a β barrel tertiary structure consisting of 12 antiparallel β strands connected by β turns. Hydrophobic residues line the core of the barrel while a large number of charged residues are present on the protein surface. A cluster of positively charged residues to one side is thought to constitute the heparin binding region of the protein [42]. The receptor binding domain is also in this vicinity but is distinct from the heparin binding region [41]. The latent instability of FGF-2 has been attributed to the significant amount of structural energy associated with the heparin binding site; binding with heparin or similar glucosaminoglycans at a ratio as low as 0.3:1 w/w (heparin:FGF-2) has been shown to stabilise the FGF-2 against trypsin-, heat-, acid- and protease-mediated inactivation [42,43,44]. In vivo, the interactions of FGF-2 with other endogenous molecules (e.g., heparin sulphate proteoglycan, heparin, fibrinogen/fibrin), presented in soluble form or bound to cell membrane, are known to control its receptor interactions, stability and concentration in an extracellular microenvironment [45].

Commercial FGF-2 is supplied as a lyophilised powder recommended to be stored at −20 °C. The lyophilisation process involves exposure of the protein to freezing, temperature ramps, negative pressure and dehydration, all of which can potentially disrupt the FGF-2 structure, encourage aggregation and ultimately lead to a reduction or loss of protein activity [46]. Lyophilisation of FGF-2 in the presence of human serum albumin, mannitol, polyethylene glycol or glucose as cryoprotectant was shown to maintain the activity of FGF-2 even when the lyophilised powder was exposed to temperatures as high as 60 °C, humidity and light for up to 10 days [47]. Other cryoprotectants, such as mannitol and lactitol, also produced a lyophilised FGF-2 product that retained functionality after 18 months storage at refrigerated or room temperatures, and was stable when exposed to 60 °C and humidity for prolonged periods of up to 30 days [48].

Clinical applications of FGF-2, e.g., administration to a wound or further formulation into a tissue engineering construct, require the lyophilised powder to be reconstituted into solutions, e.g., by the addition of water. It is this solubilized FGF-2 that is highly vulnerable to aggregation and precipitation, resulting in rapid loss of biological function [42]. The presence of two exposed thiol groups on the FGF-2 surface promotes the formation of disulphide-linked multimers, and this propensity to form disulphide bonds drives FGF-2 to form aggregates. Interestingly, aggregation in the presence of glucosaminoglycans appeared to preserve the native FGF-2 conformation while the absence of glucosaminoglycans led to a denatured conformation similar to those induced by heat or chaotrope exposure [42].

Not surprisingly, the manufacturer’s recommendations are that the reconstituted FGF-2 solutions are stable for up to 12 months only when stored at −20 °C or lower. The reconstituted FGFs solutions are stable for only about a week at 4 °C, and recommended to be used within 24 h when at ambient temperatures (<25 °C). Despite these recommendations, FGF-2 solutions at a concentration of 72 μg/mL have been shown to lose 50% functionality after just 46 min at 25 °C [49]. The functional half-life was decreased to 37, 33 and 10 min, respectively, as the storage temperature was increased to 37 °C, 42 °C and 50 °C. These findings by Shah [49] are consistent with those reported by Chen et al. [50]. The loss of functionality of the reconstituted FGF-2 solutions may be arrested by heparin, as demonstrated by Chen et al. for a 10 ng/mL FGF-2 solution co-incubated at 37 °C with a 10-fold higher concentration (100 ng/mL) of heparin [50].

The thermal- and heparin-dependent lability of the FGF-2 molecule poses major challenges for the development of functional and acceptable medicinal products. FGF-2 lyophilised with a cryoprotectant (e.g., glycine) is stable for up to 12 months storage at 4 °C, and for up to 3 weeks at room temperature (<25 °C). Lyophilisation facilitates the storage, shipping and transportation of FGF-2, but does little to mitigate its inherent instability once the lyophilised FGF-2 is reconstituted into solutions. Heparin is a good FGF-2 stabiliser; however, the inclusion of heparin is often not desirable in clinical applications because heparin has anticoagulant activity and is not an inert pharmaceutical excipient. The storage of FGF-2 medicinal solutions at −20 °C is not a practical solution to ensure product stability in clinical settings. Other alternatives, e.g., to reconstitute the lyophilised FGF-2 only when required, and applying a therapeutic regimen that requires the daily administration of multiple doses of FGF-2 to maintain pharmacological activity in vivo, are achievable only with highly compliant patients. Lou et al. found 31.3% of participants to be non-compliant with FGF-2 dosing instructions in a clinical trial on TM repair, and the tendency of these patients to administer higher than the recommended number of drops of FGF-2 solution led to liquid accumulation in the ear, with 66.7% of the patients subsequently developing secondary otorrhoea [22]. The employment of unstable FGF-2 solutions to fabricate medicinal products, e.g., tissue engineering constructs, is also not desirable. To compensate for the rapid decline in protein functionality during the manufacturing process, FGF-2 would be required to be loaded at significantly higher quantity in order to achieve the desired loading of functional FGF-2 in the final product. The associated costs and safety implications would not be acceptable to both the manufacturer and the regulatory authorities. There is, therefore, strong impetus to develop effective and practical stabilisation strategies for FGF-2 aqueous solutions to realise the clinical applications of FGF-2.

## 3. Strategies for Preserving FGF-2 Functionality in Aqueous Solutions

Many approaches have been reported in the literature for preserving the biological activity of FGF-2. Most of these efforts were directed at sustaining FGF-2 activity for cell culture research, with a smaller number of studies seeking to develop sustained release formulations of FGF-2 for tissue engineering applications. For the purposes of this review, the stabilisation approaches are classified into the broad classes of ionic interaction modification with excipients, chemical modification, and physical barrier strategies.

### 3.1. Modulating Ionic Interactions in Solutions

The addition of excipients to aqueous solutions of FGF-2 are among the simplest methods for FGF-2 stabilisation. Common strategies include the complexation of FGF-2 with its endogenous stabiliser, heparin, or heparin-like polymers, or polycations [51,52,53]. Ionic interactions between FGF-2 and the additives reduce the structural energy at the heparin-binding site, stabilise the FGF-2 native conformation and prolong its bioactivity in aqueous media [42,43,44]. Successful examples are given in Table 1, many of which demonstrated prolonged bioactivity of FGF-2 following the addition of excipients when compared with solutions of free FGF-2.

#### 3.1.1. Conjugation to Heparin and Heparin-Like Molecules

The conjugation of FGF-2 with heparin is a popular biomimicry approach underscored by the stabilising effect of heparin on endogenous FGF-2. This approach also leverages on heparin providing synergistic action on cell growth [54]. However, there are safety concerns. Pharmaceutical-grade heparins are isolated from porcine intestines and bovine lung tissues. Not only are the heparins susceptible to batch-to-batch variability [55], they can induce immune responses [56] or be contaminated or adulterated by natural and synthetic heparinoids that have led to anaphylactoid type responses and death [55]. Additionally, natural heparin is susceptible to degradation and desulfation by heparinases [57], which may adversely affect its effectiveness at stabilising FGF-2. Fractionation of heparin overcomes some of these difficulties; however, the fractionation process adds to the cost of manufacture and still does not resolve the issue of heterogeneity associated with natural heparins, which can lead to biosimilar batches not necessarily being biochemically or pharmacologically equivalent [58].

An alternative approach has been to conjugate FGF-2 with synthetic heparin-like molecules. There are a number of synthetic heparin-mimicking compounds, including sulfonated and sulphated polymers (e.g., suramin and poly(2-acrylamido-2-methyl-1-propanesulfonic acid), various polysaccharides (e.g., chitosan, cellulose and alginate), sulfonated dextrans (e.g., β-cyclodextrin tetradecasulfate) and some peptides (e.g., sulphated homooligomers of tyrosine) [59,60,61,62,63,64,65,66]. Like natural heparins, the heparin-mimics are able to stabilise proteins, stimulate cellular processes and provide anticoagulant activity [67,68,69]. Compared to heparin, however, the synthetic materials are more homogeneous in composition and less likely to undergo desulfation in vivo. The covalent conjugation of heparin-mimicking molecules to FGF-2 has therefore been a popular approach for the stabilisation of FGF-2.

A recent study conducted by Paluck et al. explored the conjugation of FGF-2 with the heparin mimetic poly(styrenesulfonate-co-poly-(ethylene glycol) methyl ether methacrylate)-b-vinyl sulfonate) (FGF2-p(SS-co-PEGMA)-b-VS) [52]. Conjugation with the p(SS-co-PEGMA) chain alone was successful in stabilising FGF-2 to heat and storage related stressors, but it required the b-vinyl sulfonate moiety to facilitate the dimerisation of the protein to FGF receptors in cells lacking heparin sulphate proteoglycans [70]. The resultant FGF-2 conjugate was able to maintain its biological activity for 7 days [52]. These are, however, in vitro cell culture data that need to be replicated in vivo, and safety data are yet to be presented. Another disadvantage is the complex methodology and involvement of noxious solvents, such as dichloromethane, which does not have generally regarded as safe (GRAS) status, in the fabrication process.

#### 3.1.2. Coacervation

Complex coacervation has been applied to FGF-2-heparin conjugates to facilitate the sustained release of FGF-2 in vivo [51]. Heparin, being negatively charged, could be condensed into a complex by interaction with a polycation, such as chitosan. Coacervation of FGF-2-heparin conjugates with a polycation (e.g., aspartic acid arginine diglyceride [PEAD]) [51] has been demonstrated to promote endothelial and mural cell proliferation and produce stable angiogenesis over 4 weeks after only a single injection into the BALB/cJ mice. Translation of the data into clinical practice will require further work to establish the safety profile of the coacervate material and to address its storage stability.

### 3.2. Chemical Modifications

The chemical approach often requires complex methodologies and the diverse range of techniques reported in the literature reflects the difficulty of stabilising the FGF-2 molecule. Methods that have been successful in prolonging the bioactivity of FGF-2 in aqueous media include point mutations of the protein and covalent grafting of the protein onto scaffold materials (Table 2) [51,52,53,54,70,71,72]. A common thread in these methods is the binding of FGF-2 to another molecule in such a way as to retain the active conformation of the FGF-2 molecule or to mask those residues of the FGF-2 molecule that contribute to its latent instability in solution. A recurring concern for this approach is the creation of novel FGF-2 compounds whose safety profiles have yet to be established in preclinical and clinical toxicology models.

#### 3.2.1. Genetic Engineering

Genetically modified FGF-2 mutants have been developed by aligning the wild-type FGF-2 protein sequence with stabilised FGF-1 mutant sequences [73], or by combining several individual stabilising mutations identified by other investigators in the field [74]. The identification of useful point mutations has been aided by computer modelling [71,75]. A recent study by Dvorak et al. used a combination of techniques to identify sequences which were important for protein functionality and those which were contributing the greatest amount of structural free energy [71]. Following an analysis of sequence conservation, mutations in regions, which might compromise protein function (such as the heparin or receptor binding sites), were avoided. Conversely, mutations that were likely to result in a significant decrease in protein free energy were promoted. The nine mutants identified in this study showed the lowest energy and, following in vitro evaluation, were found to significantly increase the functional half-life of FGF-2 from 10 h to greater than 20 days at 37 °C. Other similar studies have developed triple and quintuple FGF-2 mutants, which also exhibited improved protein stability and activity in cell culture milieu [73,74]. A very recent study [75] focuses on the hyperstable FGF-2 variants, FGF2-STABS, which were able to provide 10–100 times lower EC_50_ values and sustained in vitro FGFR-mediated activities due to increased thermal stability and lower heparin affinity in comparison to wild type FGF-2. However, this study also highlighted an important difference, with the wild type FGF-2 showing a sigmoidal dose–response profile, while the FGF2-STABS showed a biphasic response, suggesting that mutants of FGF-2 require careful preclinical evaluation before they can progress to clinical testing. A major concern with the genetically modified FGF-2s is safety, as the deleterious in vivo molecular effects of mutated proteins are well known. These may range from alterations in protein folding (e.g., sickle cell anaemia) and stability [76] to complete dysfunction (Tay–Sachs disease) [77] or a lack of regulation. For growth factors like FGF-2, a lack of regulation may lead to cellular overgrowth and malignancy in vivo [45]. The safety profiles of the novel FGF-2 mutants, as yet unknown, will have to be established in appropriate animal and human models before any consideration of clinical translation.

#### 3.2.2. Chemical Conjugation to Inorganic Materials

Chemical conjugation of FGF-2 to inorganic materials has been applied to construct functional tissues for bone repair. Tissue engineering of bone presents a unique problem. Due to the force applied to these structures, the durability of biomaterials is critical to treatment success. The physical entrapment of FGF-2 into polymer scaffolds has been shown to stabilise the FGF-2 adequately to promote osteogenesis, both in vitro and in vivo [78,79,80]. However, the organic scaffold materials showed a tendency to develop mechanical weaknesses in vivo, which then led to variable FGF-2 release [81,82]. To overcome this, Moon et al. employed inorganic materials to construct the scaffold. They prepared FGF-2-coated biphasic calcium phosphate granules using covalent bonding techniques [72], and the resultant product was found to promote alkaline phosphatase activity, a marker for osteogenic differentiation, to a higher level than free FGF-2. The promising data warrants further development. The study was performed in cell cultures and the FGF-2-conjugated calcium phosphate granules are yet to be shown to have improved durability in vivo compared to the other FGF-2 impregnated biomaterials. The release profile of FGF-2 from the granules was also not determined in the study, and the use of hexane, a potentially harmful organic solvent, for the preparation of the granules is a concern for clinical translation.

### 3.3. Physical Barrier Strategies

Physical approaches to stabilising FGF-2 are aimed at providing a barrier to protect the protein against inactivation stressors. Common strategies include the encapsulation of FGF-2 within a polymer matrix and the fabrication of a mix of FGF-2 and polymers into hydrogels or other composite scaffolds [83,84,85,86,87]. Successful examples are given in Table 3, many of which also provided for a prolonged release of FGF-2 to further sustain its biological activity. Compared to the chemical approaches, the physical stabilisation methods employed simpler methodologies and were less likely to use noxious organic solvents and novel materials of unknown safety profiles.

#### 3.3.1. Entrapment in Hydrogel Scaffolds

Hydrogels, such as hyaluronic acid, alginate, and PEG, are biocompatible materials that can be engineered to exhibit specific biodegradation profiles and binding strengths to provide good tissue tolerance and retention [89]. They have been successfully applied as carrier materials for cell transplantation, tissue engineering scaffolds and drug depots for wound and tissue regeneration. The main difficulty with hydrogels is that the liquid hydrogel formulations tend to be highly viscous and therefore difficult to administer [89,90].

Gelatin is one of the most widely applied hydrogels for fabricating FGF-2-loaded scaffolds [16,91,92,93,94]. Gelatin is derived from animal and its safety profile is well established, having been used as a pharmaceutical excipient for a long time. A gelatin hydrogel scaffold constructed by Layman et al. to control FGF-2 bioavailability at the application site [85] showed release of 80% of the FGF-2 load and a marked improvement in ischaemic limb reperfusion in a murine critical limb ischemic model over a period of 4 weeks. A sustained release of the FGF-2 load is desirable to prolong the protein bioactivity, since FGF-2 once released from the scaffold matrix into the surrounding aqueous medium is rapidly inactivated. Gelatin has also been shown to stabilise FGF-2 through ionic interactions, making it an attractive component for the formulation of stable and acceptable FGF-2 medicinal products [95,96].

#### 3.3.2. Microencapsulation

Encapsulation of FGF-2 into polymer microspheres provides a physical barrier that not only protects the protein from acid-, trypsin-, and protease-mediated inactivation, but also controls the rate of protein release to prolong its bioactivity [42,89,97]. Many of the polymers used in the production of microspheres are also hydrogels. The fabrication of the hydrogels into microspheres small enough to pass through a catheter can help overcome the difficulty associated with injecting highly viscous hydrogel liquid preparations [89,97]. The geometry of microspheres also enables them to withstand a greater mechanical strain than liquid/semi-solid hydrogels, making them an attractive option for the treatment of muscles and joints where the mechanical strain is particularly high [97].

FGF-2-loaded microspheres have also been fabricated with combinations of polymers to tailor the FGF-2 release kinetics for specific applications [98,99]. In one study, collagen was paired with alginate, which has a slower biodegradation rate, to fabricate a scaffold capable of providing a controlled release of bioactive FGF-2 for 7 days at 37 °C as determined by dissolution in DMEM, a cell culture medium [83]. The bioactivity of the microspheres in aqueous media was dependent on the alginate:collagen weight ratios, with higher collagen content associated with shorter sustained bioactivity. Combining poly(lactic-*co*-glycolic acid) (PLGA) and poly(vinyl alcohol) (PVA) to produce FGF-2 loaded microspheres has also been shown to sustain the release and stability of FGF-2 for up to 4 days in a culture of human embryonic stem cells [86]. These results and the readily customisable nature of the microsphere formulation are very promising for tissue engineering applications. Future feasibility studies will, however, have to include FGF-2 stability during the manufacturing process, as well as stability of the final microsphere formulation at ambient, refrigerated and body temperatures.

#### 3.3.3. Adsorption

The FGF-2 molecule contains highly ionised ligand and receptor binding sites, which contribute to high structural energy and the latent instability of the protein [42]. Adsorption of FGF-2 to a surface via charge interactions aids in the structural stabilisation of the protein, reduces the likelihood of spontaneous unfolding, and also provides for a sustained release of bioactive protein at the administrative site. The appropriate adsorbent would be negatively charged and acidic in nature to match the net positive charge and basic properties of FGF-2. An example is the highly porous scaffold fabricated by Yoon et al. using glass nanoparticles and a polymer that achieved a high FGF-2 adsorption efficiency, continuous release of the FGF-2 load over 4 weeks, and sustained biological activity in mesenchymal stem cell cultures for 3 weeks [87]. Comparatively, Fukunaga et al., who adsorbed FGF-2 onto the aluminium salt of cyclodextrin sulphate, observed a shorter 24-h stability of the FGF-2 against pepsin- and chromotrypsin-mediated degradation [88]. These formulations offer potential if the results can be replicated in vivo. A concern is FGF-2 desorption from the carrier material, which is influenced by the ionic strength of the surrounding medium, with the presence of positively charged ions appearing to have the greatest impact on the desorption rate [100]. The implications of this will have to be examined in vivo, where the ionic strength of biological fluids varies between 0.1 and 0.3 mol dm^−3^ [101].

## 4. Discussion

The stability of FGF-2, in particular in aqueous milieu, is widely accepted to be a major concern for the development of useful medicinal products. We have reviewed a diverse range of stabilisation strategies for FGF-2 that are reported in the literature, some of which have shown very promising data. However, there is no known published systematic stability study for FGF-2 medicinal products. Most of the stabilisation studies have a narrow scope, focusing either on cell culture alone or in conjunction with animal studies. The thermal stability of FGF-2 has not been widely studied outside of cell culture conditions. As a result, the stability and biological activity data for FGF-2 have, in the main, been obtained at 37 °C in aqueous media. FGF-2 stability profiles over the wider temperature range encountered in medicinal product manufacture, transport, storage and use have been neglected. Similarly, factors other than temperature, such as pH, proteolytic enzymes, ionic strengths, that are known to affect protein stability, have not been reported for FGF-2. Such information have implications on the applicability of FGF-2 products, e.g., as during the stages of wound healing, the pH and composition of the extracellular matrix can change [102]. It is also vital to consider the medicinal product manufacture cycle and determine whether any of the manufacturing processing conditions could affect FGF-2 stability. Regulatory authorisation of FGF-2 medicinal products require the consideration of both raw material and formulated product stability before, during, and after manufacture, including long term storage and transport considerations. None of the reviewed studies had monitored the loss of FGF-2 functionality as a result of the manufacturing process, despite the known instability of FGF-2 in solution, and the exposure of FGF-2 to aqueous milieu during the manufacture process. The lack of comprehensive published studies of this nature may well represent a substantial gap in current knowledge, which would have to be addressed to develop viable FGF-2 products for wider tissue engineering applications.

There is no “one size fits all” approach to protein stabilisation, as evidenced by the variety of techniques described in the literature. Although innovative, many investigative FGF-2 medicinal products have limited clinical translatability due to safety concerns, complex fabrication methodologies, which would make commercial upscaling of the product difficult and costly, and/or a lack of comprehensive efficacy studies. Despite its inherent instability, FGF-2 has been shown to enhance cellular proliferation, migration, differentiation, and promote angiogenesis in a wide variety of tissues [12,13,14]. Therefore, once the stability issues preventing the successful incorporation of FGF-2 into safe and effective medicinal products can be overcome, the potential clinical applications for FGF-2 will likely be many and varied.

## Figures and Tables

**Table 1:** Examples of aqueous environment modification techniques, which have demonstrated increased stability and/or bioactivity of the modified fibroblast growth factor (FGF)-2 compared with the unmodified FGF-2.

Strategy	Technique	Comparative Stability	Potential Application	Model for Functional Assessment	Ref
Conjugation with a heparin-mimicking polymer	Conjugation with p(SS-co-PEGMA)-b-VS^1^, which contains a segment that enhances FGF-2 stability and another that binds FGF-2 receptor.	Biological activity extended to 7 days at 4 °C and 23 °C. Facilitated binding to receptor and increased migration of endothelial cells by 80% compared to free FGF-2.	Tissue engineering and wound healing	Human dermal fibroblasts Human umbilical vein endothelial cells	[52]
Complexation with heparin	Complexation with heparin immobilised on glass/silicone slides	Maintained bioactivity for up to 12 h at 37 °C. Rate of cell migration increased by 14 µm/h for vascular smooth muscle cells compared to free FGF-2	Tissue engineering and wound healing	Human vascular smooth muscle cells, endothelial cells and Mesenchyme stem cells	[53]
Complex coacervation	Coacervation in the presence of a polycation (PEAD)^2^ and heparin	Maintained in vivo bioactivity for over 4 weeks from a single injection. Increased proliferation of endothelial and mural cells by 47% at 37 °C, when compared with free FGF-2.	Tissue engineering	BALB/cJ mice, murine endothelial and mural cells	[51]

^1^ Poly(styrene sulfonate-co-poly-(ethylene glycol) methyl ether methacrylate)-b-vinyl sulfonate). ^2^ Aspartic acid arginine diglyceride.

**Table 2:** Examples of chemical modification techniques, which have demonstrated increased stability and/or bioactivity of the modified FGF-2 compared with the unmodified FGF-2.

Strategy	Technique	Comparative Stability	Potential Application	Model for Functional Assessment	Ref
Genetic engineering	Nine-point mutant of a low molecular weight isoform of FGF-2	In vitro functional half-life at 37 °C improved from 10 h to more than 20 days.	Cell culture research	Human embryonic stem cells	[71]
Covalent grafting	Conjugation onto biphasic calcium phosphate scaffolds ^1^	Increase alkaline phosphatase activity at 37 °C by up to 200% when compared with free FGF-2.	Tissue engineering	Human mesenchyme stem cells	[72]

^1^ 70% hydroxyapatite coated with 30% *β*-tricalcium phosphate.

**Table 3:** Examples of physical protective methods to stabilise and/or prolong the biological activity of FGF-2.

Strategy	Technique	Comparative Stability	Potential Application	Model for Functional Assessment	Ref
Entrapment within hydrogel scaffolds	Gelatin-polylysine and Gelatin-polyglutamic acid hydrogels loaded with FGF-2	FGF-2 release was sustained over 28 days at 37 °C. Limb reperfusion and angiogenesis was 69.3% higher in the hydrogel treated mice than in mice treated with free FGF-2.	Tissue engineering	Human neo-natal fibroblasts, BALB/c mice	[85]
Entrapment within polymer microspheres	FGF-2, magnesium hydroxide, heparin, PLGA and PVA^1^ were mixed to create a water/oil/water emulsion, from which microspheres were fabricated.	FGF-2 levels sustained for 3-4 days at 37 °C, compared with no activity remaining at 24 h for free FGF-2.	Cell culture research	Human embryonic stem cells, human neural stem cells, induced pluripotent stem cells	[86]
	Alginate:collagen microspheres loaded with FGF-2	Sustained release over 7 days at 37 °C	Tissue engineering	C57/Bl6 mice, porcine aortic endothelial cells	[83]
Entrapment within polyelectrolyte polymer	HEMA^2^ hydrogel was loaded with either FGF-2 or FGF-2 and heparin.	Hydrogel containing only FGF-2 released FGF-2 over 6 days at 37 °C. Addition of heparin to the matrix prolonged the FGF-2 release to 12 days	Tissue engineering	Neural stem cells	[84]
Ionic entrapment within biodegradable multilayer thin film	Thin film consisting of 10–50 tetralayers made up of beta aminoester/ heparin/FGF-2/ heparin.	Up to 5 days sustained release of FGF-2 from the film, with in vitro biological activity sustained for 12 days at 37 °C.	Tissue engineering or wound healing	Murine MC3T3 pre-osteoblast cells	[54]
Adsorption onto polymer carriers	Bioactive glass nanoparticles were fabricated with the biodegradable polylactic acid into highly porous scaffolds. FGF-2 was then adsorbed onto the scaffold surface.	FGF-2 release was sustained over 4 weeks at 37 °C with an almost linear release pattern after the initial 3 days. Biological effect was observed over 3 weeks.	Tissue engineering	Mesenchymal stem cells	[87]
	FGF-2 adsorbed onto the aluminium salt of cyclodextrin sulphate	FGF-2 retained activity following exposure to pepsin for 6 h or chromotrypsin for 24 h. Showed 62% improved efficacy against gastric ulcers in a rat model compared to 15% improvement with free FGF-2.	Wound healing	BHK-21 cells, Male Jal:Wistar rats	[88]

^1^ Polyvinyl alcohol; ^2^ Polyelectrolyte-modified hydroxyethyl methacrylate.

## References

[1] Kawai K., Suzuki S., Tabata Y., Ikada Y., Nishimura Y. (2000). Accelerated tissue regeneration through incorporation of basic fibroblast growth factor-impregnated gelatin microspheres into artificial dermis. Biomaterials.

[2] Gao Y., Zhu S., Luo E., Li J., Feng G., Hu J. (2009). Basic fibroblast growth factor suspended in Matrigel improves titanium implant fixation in ovariectomized rats. J. Control. Release.

[3] Takafuji H., Suzuki T., Okubo Y., Fujimura K., Bessho K. (2007). Regeneration of articular cartilage defects in the temporomandibular joint of rabbits by fibroblast growth factor-2: A pilot study. Int. J. Oral Maxillofac. Surg..

[4] Hankemeier S., Keus M., Zeichen J., Jagodzinski M., Barkhausen T., Bosch U., Krettek C., Van Griensven M. (2005). Modulation of proliferation and differentiation of human bone marrow stromal cells by fibroblast growth factor 2: Potential implications for tissue engineering of tendons and ligaments. Tissue Eng..

[5] Kasai M., Jikoh T., Fukumitsu H., Furukawa S. (2014). FGF-2-responsive and spinal cord-resident cells improve locomotor function after spinal cord injury. J. Neurotrauma.

[6] Gospodarowicz D. (1975). Purification of a fibroblast growth factor from bovine pituitary. J. Biol. Chem..

[7] Fox G.M. (1988). Production, biological activity, and structure of recombinant basic fibroblast growth factor and an analog with cysteine replaced by serine. J. Biol. Chem..

[8] Plouët J., Schilling J., Gospodarowicz D. (1989). Isolation and characterization of a newly identified endothelial cell mitogen produced by AtT-20 cells. EMBO J..

[9] Abraham J.A., Whang J.L., Tumolo A., Mergia A., Friedman J., Gospodarowicz D., Fiddes J.C. (1986). Human basic fibroblast growth factor: Nucleotide sequence and genomic organization. EMBO J..

[10] Simpson R.J., Moritz R.L., Lloyd C.J., Fabri L.J., Nice E.C., Rubira M.R., Burgess A.W. (1987). Primary structure of ovine pituitary basic fibroblast growth factor. FEBS Lett..

[11] Shimasaki S., Emoto N., Koba A., Mercado M., Shibata F., Cooksey K., Baird A., Ling N. (1988). Complementary DNA cloning and sequencing of rat ovarian basic fibroblast growth factor and tissue distribution study of its mRNA. Biochem. Biophys. Res. Commun..

[12] Beenken A., Mohammadi M. (2009). The FGF family: Biology, pathophysiology and therapy. Nat. Rev. Drug Discov..

[13] Yun Y.-R., Won J.E., Jeon E., Lee S., Kang W., Jo H., Jang J.-H., Shin U.S., Kim H.-W. (2010). Fibroblast growth factors: Biology, function, and application for tissue regeneration. J. Tissue Eng..

[14] Park J.W., Hwang S.R., Yoon I.S. (2017). Advanced growth factor delivery systems in wound management and skin regeneration. Molecules.

[15] Xuan X., Zhou Y., Chen A., Zheng S., An Y., He H., Huang W., Chen Y., Yang Y., Li S. (2020). Silver crosslinked injectable bFGF-eluting supramolecular hydrogels speed up infected wound healing. J. Mater. Chem. B.

[16] Washio A., Teshima H., Yokota K., Kitamura C., Tabata Y. (2019). Preparation of gelatin hydrogel sponges incorporating bioactive glasses capable for the controlled release of fibroblast growth factor-2. J. Biomater. Sci. Polym. Ed..

[17] Kanemaru S.-I., Umeda H., Kitani Y., Nakamura T., Hirano S., Ito J. (2011). Regenerative treatment for tympanic membrane perforation. Otol. Neurotol..

[18] Lou Z., Huang P., Yang J., Xiao J., Chang J. (2016). Direct application of bFGF without edge trimming on human subacute tympanic membrane perforation. Am. J. Otolaryngol..

[19] Lou Z., Tang Y., Wu X. (2012). Analysis of the effectiveness of basic fibroblast growth factor treatment on traumatic perforation of the tympanic membrane at different time points. Am. J. Otolaryngol..

[20] Lou Z., Wang Y. (2015). Evaluation of the optimum time for direct application of fibroblast growth factor to human traumatic tympanic membrane perforations. Growth Factors.

[21] Lou Z., Wang Y., Yu G. (2014). Effects of basic fibroblast growth factor dose on traumatic tympanic membrane perforation. Growth Factors.

[22] Lou Z.-C., Lou Z.-H. (2018). The short- and long-term adverse effects of FGF-2 on tympanic membrane perforations. Acta Otorhinolaryngol. Ital..

[23] Lou Z.C., He J.G. (2011). A randomised controlled trial comparing spontaneous healing, gelfoam patching and edge-approximation plus gelfoam patching in traumatic tympanic membrane perforation with inverted or everted edges. Clin. Otolaryngol..

[24] Lou Z.C., Wang Y.B.Z. (2013). Healing outcomes of large (>50%) traumatic membrane perforations with inverted edges following no intervention, edge approximation and fibroblast growth factor application; a sequential allocation, three-armed trial. Clin. Otolaryngol..

[25] Acharya A.N., Coates H., Tavora-Vièira D., Rajan G.P. (2015). A pilot study investigating basic fibroblast growth factor for the repair of chronic tympanic membrane perforations in pediatric patients. Int. J. Pediatr. Otorhinolaryngol..

[26] Chen J.-H. (2016). Comparison of FGF-2, FLOX, and gelfoam patching for traumatic tympanic membrane perforation. Otol. Neurotol..

[27] Hakuba N., Hato N., Okada M., Mise K. (2015). Preoperative factors affecting tympanic membrane regeneration therapy using an atelocollagen and basic fibroblast growth factor. JAMA Otolaryngol. Head Neck Surg..

[28] Hakuba N., Iwanaga M., Tanaka S., Hiratsuka Y., Kumabe Y., Konishi M., Okanoue Y., Hiwatashi N., Wada T. (2010). Basic fibroblast growth factor combined with atelocollagen for closing chronic tympanic membrane perforations in 87 patients. Otol. Neurotol..

[29] Omae K., Kanemaru S.-I., Nakatani E., Kaneda H., Nishimura T., Tona R., Naito Y., Kawamoto A., Fukushima M. (2017). Regenerative treatment for tympanic membrane perforation using gelatin sponge with basic fibroblast growth factor. Auris Nasus Larynx.

[30] Matsumine H., Fujimaki H., Takagi M., Mori S., Iwata T., Shimizu M., Takeuchi M. (2019). Full-thickness skin reconstruction with basic fibroblast growth factor-impregnated collagen-gelatin sponge. Regen. Ther..

[31] Lou Z.C., Lou Z.H., Zhang Q.P. (2012). Traumatic tympanic membrane perforations: A study of etiology and factors affecting outcome. Am. J. Otolaryngol..

[32] Lou Z.C. (2012). Spontaneous healing of traumatic eardrum perforation: Outward epithelial cell migration and clinical outcome. Otolaryngol. Head Neck Surg..

[33] Lou Z., Yang J., Tang Y., Xiao J. (2015). Risk factors affecting human traumatic tympanic membrane perforation regeneration therapy using fibroblast growth factor-2. Growth Factors.

[34] Lou Z.C., Lou Z.H., Liu Y.C., Chang J. (2016). Healing human moderate and large traumatic tympanic membrane perforations using basic fibroblast growth factor, 0.3% ofloxacin eardrops, and gelfoam patching. Otol. Neurotol..

[35] Lou Z., Lou Z., Tang Y., Xiao J. (2016). The effect of ofloxacin otic drops on the regeneration of human traumatic tympanic membrane perforations. Clin. Otolaryngol..

[36] Morimoto N., Yoshimura K., Niimi M., Ito T., Aya R., Fujitaka J., Tada H., Teramukai S., Murayama T., Toyooka C. (2013). Novel collagen/gelatin scaffold with sustained release of basic fibroblast growth factor: Clinical Trial for chronic skin ulcers. Tissue Eng. Part A.

[37] Burgess W.H., Maciag T. (1989). The Heparin-binding (fibroblast) growth factor family of proteins. Annu. Rev. Biochem..

[38] Andreopoulos F.M., Persaud I. (2006). Delivery of basic fibroblast growth factor (bFGF) from photoresponsive hydrogel scaffolds. Biomaterials.

[39] Cai S., Liu Y., Zheng Shu X., Prestwich G.D. (2005). Injectable glycosaminoglycan hydrogels for controlled release of human basic fibroblast growth factor. Biomaterials.

[40] Freudenberg U., Hermann A., Welzel P.B., Stirl K., Schwarz S.C., Grimmer M., Zieris A., Panyanuwat W., Zschoche S., Meinhold D. (2009). A star-PEG–heparin hydrogel platform to aid cell replacement therapies for neurodegenerative diseases. Biomaterials.

[41] Zhang J.D., Cousens L.S., Barr P.J., Sprang S.R. (1991). Three-dimensional structure of human basic fibroblast growth factor, a structural homolog of interleukin 1 beta. Proc. Natl. Acad. Sci. USA.

[42] Wang J., Shahrokh Z., Vemuri S., Eberlein G., Beylin I., Busch M., Pearlman R., Wang J.Y. (2002). Characterization, stability, and formulations of basic fibroblast growth factor. Formulation, Characterization, and Stability of Protein Drugs: Case Histories.

[43] Vemuri S., Beylin I., Sluzky V., Stratton P.A., Eberlein G.A., Wang Y.J. (1994). The stability of bFGF against thermal denaturation. J. Pharm. Pharmacol..

[44] Furue M.K., Na J., Jackson J.P., Okamoto T., Jones M., Baker D., Hata R.-I., Moore H.D., Sato J.D., Andrews P.W. (2008). Heparin promotes the growth of human embryonic stem cells in a defined serum-free medium. Proc. Natl. Acad. Sci. USA.

[45] Akl M.R., Nagpal P., Ayoub N.M., Tai B., Prabhu S.A., Capac C.M., Gliksman M., Goy A., Suh K.S. (2016). Molecular and clinical significance of fibroblast growth factor 2 (FGF2 /bFGF) in malignancies of solid and hematological cancers for personalized therapies. Oncotarget.

[46] Ohtake S., Kita Y., Arakawa T. (2011). Interactions of formulation excipients with proteins in solution and in the dried state. Adv. Drug Deliv. Rev..

[47] Fang H.Z., Zheng Z.S., Zhu A.T., Lan X., Huang X.K. (2012). Recombinant Bovine Basic Fibroblast Growth Factor Freeze-Dried Formulation for External Application. CN Patent.

[48] Fang H.Z., Zheng Z.S., Zhu A.T., Lan X., Huang X.K. (2016). External Recombinant Bovine Basic Fibroblast Growth Factor Lyophilized Preparation. CN Patent.

[49] Shah D. (1998). Effect of Various Additives on the Stability of Basic Fibroblast Growth Factor and Development of an Intradermal Injectable Formulation. Ph.D. Thesis.

[50] Chen G., Gulbranson D.R., Yu P., Hou Z., Thomson J.A. (2012). Thermal stability of fibroblast growth factor protein is a determinant factor in regulating self-renewal, differentiation, and reprogramming in human pluripotent stem cells. Stem Cells.

[51] Chu H., Gao J., Chen C.-W., Huard J., Wang Y. (2011). Injectable fibroblast growth factor-2 coacervate for persistent angiogenesis. Proc. Natl. Acad. Sci. USA.

[52] Paluck S.J., Nguyen T.H., Lee J.P., Maynard H.D. (2016). A heparin-mimicking block copolymer both stabilizes and increases the activity of fibroblast growth factor 2 (FGF2). Biomacromolecules.

[53] Wu J., Mao Z., Hong Y., Han L., Gao C. (2013). Conjugation of basic fibroblast growth factor on a heparin gradient for regulating the migration of different types of cells. Bioconj. Chem..

[54] Macdonald M.L., Rodriguez N.M., Shah N.J., Hammond P.T. (2010). Characterization of tunable FGF-2 releasing polyelectrolyte multilayers. Biomacromolecules.

[55] Liu H., Zhang Z., Linhardt R.J. (2009). Lessons learned from the contamination of heparin. Nat. Prod. Rep..

[56] Warkentin T.E., Sheppard J.-A.I., Moore J.C., Cook R.J., Kelton J.G. (2009). Studies of the immune response in heparin-induced thrombocytopenia. Blood.

[57] Hovingh P., Linker A. (1970). The enzymatic degradation of heparin and heparitin sulfate: III. Purification of a heparitinase and a heparinase from flavobacteria. J. Biol. Chem..

[58] Merli G.J., Groce J.B. (2010). Pharmacological and clinical differences between low-molecular-weight heparins: Implications for prescribing practice and therapeutic interchange. Pharm. Ther..

[59] Papy-Garcia D., Barbier-Chassefière V., Rouet V., Kerros M.-E., Klochendler C., Tournaire M.-C., Barritault D., Caruelle J.-P., Petit E. (2005). Nondegradative sulfation of polysaccharides. Synthesis and structure characterization of biologically active heparan sulfate mimetics. Macromolecules.

[60] Mauzac M., Jozefonvicz J. (1984). Anticoagulant activity of dextran derivatives. Part I: Synthesis and characterization. Biomaterials.

[61] Mammadov R., Mammadov B., Guler M.O., Tekinay A.B. (2012). Growth factor binding on heparin mimetic peptide nanofibers. Biomacromolecules.

[62] Coombe D.R., Kett W.C. (2012). Heparin mimetics. Handb. Exp. Pharmacol..

[63] Hassan H.H. (2007). Chemistry and biology of heparin mimetics that bind to fibroblast growth factors. Mini Rev. Med. Chem..

[64] Kim S.H., Kiick K.L. (2007). Heparin-mimetic sulfated peptides with modulated affinities for heparin-binding peptides and growth factors. Peptides.

[65] Logeart-Avramoglou D., Jozefonvicz J. (1999). Carboxymethyl benzylamide sulfonate dextrans (CMDBS), a family of biospecific polymers endowed with numerous biological properties: A review. J. Biomed. Mater. Res..

[66] Maynard H.D., Hubbell J.A. (2005). Discovery of a sulfated tetrapeptide that binds to vascular endothelial growth factor. Acta Biomater..

[67] Tardieu M., Gamby C., Avramoglou T., Jozefonvicz J., Barritault D. (1992). Derivatized dextrans mimic heparin as stabilizers, potentiators, and protectors of acidic or basic FGF. J. Cell. Physiol..

[68] Liekens S., Leali D., Neyts J., Esnouf R., Rusnati M., Dell’Era P., Maudgal P.C., De Clercq E., Presta M. (1999). Modulation of fibroblast growth factor-2 receptor binding, signaling, and mitogenic activity by heparin-mimicking polysulfonated compounds. Mol. Pharmacol..

[69] Nguyen T.H., Paluck S.J., McGahran A.J., Maynard H.D. (2015). Poly(vinyl sulfonate) Facilitates bFGF-Induced Cell Proliferation. Biomacromolecules.

[70] Nguyen T.H., Kim S.-H., Decker C.G., Wong D.Y., Loo J.A., Maynard H.D. (2013). A heparin-mimicking polymer conjugate stabilizes basic fibroblast growth factor. Nat. Chem..

[71] Dvorak P., Bednar D., Vanacek P., Balek L., Eiselleova L., Stepankova V., Sebestova E., Kunova Bosakova M., Konecna Z., Mazurenko S. (2017). Computer-assisted engineering of hyperstable fibroblast growth factor 2. Biotechnol. Bioeng..

[72] Moon K.-S., Choi E.-J., Oh S., Kim S. (2015). The Effect of Covalently Immobilized FGF-2 on Biphasic Calcium Phosphate Bone Substitute on Enhanced Biological Compatibility and Activity. BioMed Res. Int..

[73] Nolle V. (2015). Fibroblast Growth Factor Muteins with Increased Activity. U.S. Patent.

[74] Jeong S.S. (2015). Thermostable Variants of Fibroblast Growth Factors. U.S. Patent.

[75] Koledova Z., Sumbal J., Rabata A., de La Bourdonnaye G., Chaloupkova R., Hrdlickova B., Damborsky J., Stepankova V. (2019). Fibroblast Growth Factor 2 Protein Stability Provides Decreased Dependence on Heparin for Induction of FGFR Signaling and Alters ERK Signaling Dynamics. Front. Cell Dev. Biol..

[76] Valastyan J.S., Lindquist S. (2014). Mechanisms of protein-folding diseases at a glance. Dis. Models Mech..

[77] Myerowitz R., Costigan F.C. (1988). The major defect in Ashkenazi Jews with Tay-Sachs disease is an insertion in the gene for the alpha-chain of beta-hexosaminidase. J. Biol. Chem..

[78] Lisignoli G., Zini N., Remiddi G., Piacentini A., Puggioli A., Trimarchi C., Fini M., Maraldi N.M., Facchini A. (2001). Basic fibroblast growth factor enhances in vitro mineralization of rat bone marrow stromal cells grown on non-woven hyaluronic acid based polymer scaffold. Biomaterials.

[79] Tanihara M., Suzuki Y., Yamamoto E., Noguchi A., Mizushima Y. (2001). Sustained release of basic fibroblast growth factor and angiogenesis in a novel covalently crosslinked gel of heparin and alginate. J. Biomed. Mater. Res..

[80] Zellin G., Linde A. (2000). Effects of recombinant human fibroblast growth factor-2 on osteogenic cell populations during orthopic osteogenesis in vivo. Bone.

[81] Jeong I., Yu H.S., Kim M.K., Jang J.H., Kim H.W. (2010). FGF2-adsorbed macroporous hydroxyapatite bone granules stimulate in vitro osteoblastic gene expression and differentiation. J. Mater. Sci. Mater. Med..

[82] Sogo Y., Ito A., Onoguchi M., Oyane A., Tsurushima H., Ichinose N. (2007). Formation of a FGF-2 and calcium phosphate composite layer on a hydroxyapatite ceramic for promoting bone formation. Biomed. Mater. (Bristol Engl.).

[83] Ali Z., Islam A., Sherrell P., Le-Moine M., Lolas G., Syrigos K., Rafat M., Jensen L.D. (2018). Adjustable delivery of pro-angiogenic FGF-2 by alginate:collagen microspheres. Biol. Open.

[84] Galderisi U., Peluso G., Di Bernardo G., Calarco A., D’Apolito M., Petillo O., Cipollaro M., Fusco F.R., Melone M.A.B. (2013). Efficient cultivation of neural stem cells with controlled delivery of FGF-2. Stem Cell Res..

[85] Layman H., Spiga M.-G., Brooks T., Pham S., Webster K.A., Andreopoulos F.M. (2007). The effect of the controlled release of basic fibroblast growth factor from ionic gelatin-based hydrogels on angiogenesis in a murine critical limb ischemic model. Biomaterials.

[86] Lotz S., Goderie S., Tokas N., Hirsch S.E., Ahmad F., Corneo B., Le S., Banerjee A., Kane R.S., Stern J.H. (2013). Sustained levels of FGF2 maintain undifferentiated stem cell cultures with biweekly feeding. PLoS ONE.

[87] Yoon J.-Y., Kim J.-J., El-Fiqi A., Jang J.-H., Kim H.-W. (2017). Ultrahigh protein adsorption capacity and sustained release of nanocomposite scaffolds: Implication for growth factor delivery systems. RSC Adv..

[88] Fukunaga K., Hijikata S., Ishimura K., Ohtani Y., Kimura K., Fuji M., Hata Y. (1996). Composition of Stabilized Fibroblast Growth Factor. U.S. Patent.

[89] Mayfield A.E., Tilokee E.L., Latham N., McNeill B., Lam B.-K., Ruel M., Suuronen E.J., Courtman D.W., Stewart D.J., Davis D.R. (2014). The effect of encapsulation of cardiac stem cells within matrix-enriched hydrogel capsules on cell survival, post-ischemic cell retention and cardiac function. Biomaterials.

[90] Gelmi A., Cieslar-Pobuda A., de Muinck E., Los M., Rafat M., Jager E.W.H. (2016). Direct mechanical stimulation of stem cells: A beating electromechanically active scaffold for cardiac tissue engineering. Adv. Healthc. Mater..

[91] Iwakura A., Tabata Y., Koyama T., Doi K., Nishimura K., Kataoka K., Fujita M., Komeda M. (2003). Gelatin sheet incorporating basic fibroblast growth factor enhances sternal healing after harvesting bilateral internal thoracic arteries. J. Thorac. Cardiovasc. Surg..

[92] Pieper J.S., Hafmans T., van Wachem P.B., van Luyn M.J.A., Brouwer L.A., Veerkamp J.H., van Kuppevelt T.H. (2002). Loading of collagen-heparan sulfate matrices with bFGF promotes angiogenesis and tissue generation in rats. J. Biomed. Mater. Res..

[93] Lee H., Lim S., Birajdar M.S., Lee S.H., Park H. (2016). Fabrication of FGF-2 immobilized electrospun gelatin nanofibers for tissue engineering. Int. J. Biol. Macromol..

[94] Tokunaga T., Arimura H., Yonemitsu R., Karasugi T., Ide J., Mizuta H. (2017). Effect of FGF-2-impregnated gelatin hydrogel sheet incorporation into the bony trough on rotator cuff healing: A rabbit model. J. Shoulder Elb. Surg..

[95] Gounis M.J., Spiga M.G., Graham R.M., Wilson A., Haliko S., Lieber B.B., Wakhloo A.K., Webster K.A. (2005). Angiogenesis is confined to the transient period of VEGF expression that follows adenoviral gene delivery to ischemic muscle. Gene Ther..

[96] Rasmussen H.S., Rasmussen C.S., Macko J. (2002). VEGF gene therapy for coronary artery disease and peripheral vascular disease. Cardiovasc. Radiat. Med..

[97] Sherrell P.C., Elmén K., Cieślar-Pobuda A., Wiecheć E., Lemoine M., Arzhangi Z., Silverå Ejneby M., Brask J., Daka J.N., Rafat M. (2016). Cardiac and stem cell-cocooned hybrid microspheres: A multi factorial design approach. Sens. Actuators B Chem..

[98] Ayala P., Caves J., Dai E., Siraj L., Liu L., Chaudhuri O., Haller C.A., Mooney D.J., Chaikof E.L. (2015). Engineered composite fascia for stem cell therapy in tissue repair applications. Acta Biomater..

[99] Khorshidi S., Solouk A., Mirzadeh H., Mazinani S., Lagaron J.M., Sharifi S., Ramakrishna S. (2015). A review of key challenges of electrospun scaffolds for tissue-engineering applications. J. Tissue Eng. Regen. Med..

[100] Tabata Y., Nagano A., Muniruzzaman M., Ikada Y. (1998). In vitro sorption and desorption of basic fibroblast growth factor from biodegradable hydrogels. Biomaterials.

[101] Bower V.E., Paabo M., Bates R. (1961). A Standard for the Measurement of the pH of Blood and Other Physiological Media. J. Res. Natl. Bur. Stand..

[102] Schneider L.A., Korber A., Grabbe S., Dissemond J. (2007). Influence of pH on wound-healing: A new perspective for wound-therapy?. Arch. Dermatol. Res..

